# The newly proposed alanine aminotransferase to high-density lipoprotein cholesterol ratio has shown effectiveness in identifying non-alcoholic fatty liver disease

**DOI:** 10.3389/fendo.2023.1239398

**Published:** 2023-08-31

**Authors:** Jiajun Qiu, Maobin Kuang, Ruijuan Yang, Changhui Yu, Shiming He, Guotai Sheng, Yang Zou

**Affiliations:** ^1^ Department of Internal Medicine, Medical College of Nanchang University, Jiangxi Provincial People's Hospital, Nanchang, Jiangxi, China; ^2^ Jiangxi Cardiovascular Research Institute, Jiangxi Provincial People’s Hospital, The First Affiliated Hospital of Nanchang Medical College, Nanchang, Jiangxi, China; ^3^ Department of Endocrinology, Jiangxi Provincial People’s Hospital, The First Affiliated Hospital of Nanchang Medical College, Nanchang, Jiangxi, China; ^4^ Jiangxi Provincial Geriatric Hospital, The First Affiliated Hospital of Nanchang Medical College, Nanchang, Jiangxi, China; ^5^ Jiangxi Provincial People’s Hospital, The First Affiliated Hospital of Nanchang Medical College, Nanchang, Jiangxi, China

**Keywords:** ALT/HDL-C ratio, alanine aminotransferase to high-density lipoprotein cholesterol ratio, non-alcoholic fatty liver disease, NAFLD, ALT to HDL-C ratio

## Abstract

**Objective:**

Alanine aminotransferase (ALT) and high-density lipoprotein cholesterol (HDL-C) are important predictive factors for non-alcoholic fatty liver disease (NAFLD). The aim of this study was to analyze the association between the ALT/HDL-C ratio and NAFLD.

**Methods:**

We conducted a retrospective analysis of data from 14,251 individuals participating in the NAGALA project’s health screening program. The presence of NAFLD was diagnosed based on the participants’ alcohol consumption status and liver ultrasonography images. Multivariable logistic regression models were used to assess the association between the ALT/HDL-C ratio and NAFLD. Receiver operating characteristic (ROC) analysis was performed to determine and compare the effectiveness of ALT, HDL-C, the aspartate aminotransferase to HDL-C (AST/HDL-C) ratio, the gamma-glutamyl transferase to HDL-C (GGT/HDL-C) ratio and the ALT/HDL-C ratio in identifying NAFLD.

**Results:**

We observed a significant positive association between the ALT/HDL-C ratio and the prevalence of NAFLD. For each standard deviation (SD) increase in the ALT/HDL-C ratio, the adjusted odds ratio (OR) for NAFLD among the participants was 3.05 [95% confidence interval (CI): 2.63, 3.53], with the highest quartile of ALT/HDL-C ratio having a 9.96-fold increased risk compared to the lowest quartile. In further subgroup analyses stratified by gender, age, and waist circumference (WC), we observed a significantly higher risk of NAFLD associated with the ALT/HDL-C ratio among individuals aged ≥45 years, males, and those who were abdominal obesity. Furthermore, based on the results of ROC analysis, we found that the ALT/HDL-C ratio [area under the curves (AUC): 0.8553] was significantly superior to ALT, HDL-C, AST/HDL-C ratio and GGT/HDL-C ratio in identifying NAFLD (All Delong *P*<0.05); the threshold of suggested ALT/HDL-C ratio for identifying NAFLD was 15.97.

**Conclusion:**

This population-based study demonstrates a positive association between the ALT/HDL-C ratio and NAFLD. The ALT/HDL-C ratio can effectively identify individuals with NAFLD.

## Introduction

NAFLD is a significant public health problem, affecting approximately one-fourth of the global adult population (1, 2). It is characterized by hepatic lipid accumulation and inflammation, leading to structural and functional damage to the liver (1, 3, 4), and exerting adverse effects on multiple organs through disturbances in intrahepatic and extrahepatic metabolic regulation (5, 6). To date, the mechanisms leading to NAFLD remain unclear. Researchers have proposed multiple mechanisms, among which insulin resistance (IR) appears to be central to the pathogenesis of NAFLD (1, 7). Additionally, it is worth noting that more and more recent studies have found that mitochondrial metabolism was involved in the pathogenesis of NAFLD in various ways, including autophagy, oxidative stress, cell division and mitochondrial quality control (8–10). These new evidences showed that mitochondrial dysfunction may play a key role in the occurrence and progression of NAFLD (8–10), which provided useful new ideas for the treatment of NAFLD. Considering the current global pandemic of NAFLD which has caused a serious disease burden to society and individuals (1–6), in addition to making a breakthrough in the pathogenesis of NAFLD as soon as possible, we also need to develop simple indicators that can efficiently and quickly identify high-risk groups of NAFLD. These efforts are crucial for the prevention, management and treatment of NAFLD.

ALT is a key enzyme that reflects liver function in both physiological and pathophysiological contexts (11). Compared to AST and GGT, ALT is the liver enzyme most closely associated with hepatic fat accumulation (12, 13) and is frequently used as a diagnostic/predictive marker for NAFLD in epidemiological studies (12–15). HDL is the smallest lipoprotein, and one of its most well-known functions is promoting reverse cholesterol transport, allowing excess cholesterol to be removed from macrophages and further excreted from the body via bile (16, 17). In its natural state, we typically monitor HDL-C levels to reflect the status of cholesterol reverse transport. The liver is a central organ responsible for lipid metabolism, and when there is increased hepatic lipid accumulation, the reverse cholesterol transport mediated by HDL and the plasma cholesterol-carrying capacity become impaired, leading to a decrease in plasma HDL-C levels and a compromised protective mechanism against atherosclerosis (17–19). Epidemiological evidence has further confirmed the significant role of low HDL-C levels as an important risk factor for NAFLD (20, 21), while certain pharmacological interventions targeting lipid proteins have provided promising prospects for NAFLD patients (3), albeit with the need to overcome potential side effects of drug treatments. The ALT/HDL-C ratio is a recently proposed composite index that, according to the study by Cao et al., significantly improved the predictive performance for diabetes compared to single parameters (22). This combination may have potential applications in metabolic diseases. Considering the strong associations between the simple and commonly used liver enzyme marker ALT and the atherosclerosis indicator HDL-C with NAFLD (12–15, 20, 21), we speculate that the combination of ALT and HDL-C ratios is closely associated with NAFLD, and this combination may further enhance the ability to identify NAFLD. To investigate this hypothesis, the current study examined the association between ALT/HDL-C ratio and NAFLD based on a large sample of adults from the NAGALA project.

## Methods

### Data source

This study conducted a post-analysis using data from the NAGALA project. The data were sourced from Murakami Memorial Hospital in the Gifu region of Japan. The available public data have been uploaded by Okamura et al. to the DRYAD database for sharing (23). According to the terms of service of the DRYAD database, we are authorized to perform post-analysis using the database’s data without infringing upon the original authors’ rights.

### Study population and design

**Figure 1:**
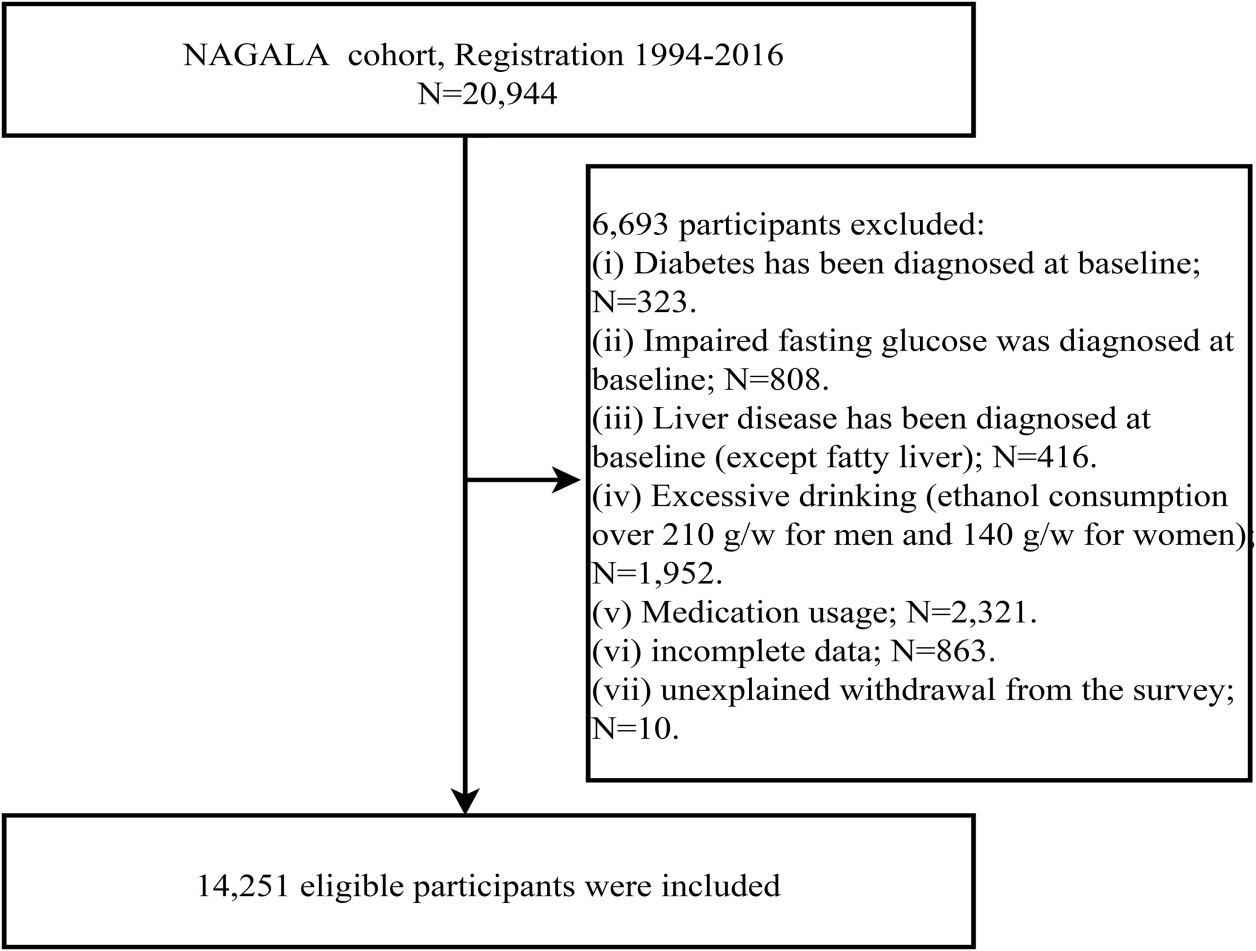
Flow chart for inclusion and exclusion of study participants.

The design of the NAGALA project has been described in detail in a previous study by Professor Okamura (24). In summary, the project was established on May 1, 1994, and included individuals undergoing health screenings at the Murakami Memorial Hospital Health Examination Center. Its aim was to investigate risk factors for chronic diseases such as diabetes and NAFLD. In Professor Okamura’s previous study, they extracted research data from a total of 20,944 participants in the NAGALA project from 1994 to 2016 and analyzed the association between ectopic fat obesity and diabetes. In the current study, we selected cross-sectional data from the NAGALA project spanning 1994 to 2016 to analyze the association between the ALT/HDL-C ratio and NAFLD. Building upon the previous study design, we excluded participants who were diagnosed with liver diseases other than fatty liver at baseline (n=416), participants who consumed alcohol at a rate of ≥210g/week for males or ≥140g/week for females at baseline (n=1,952) (25), participants diagnosed with diabetes at baseline (n=323) or with a fasting plasma glucose (FPG) level exceeding 6.1mmol/L at baseline (n=808), participants with incomplete baseline data (n=863), participants taking oral medication at baseline (n=2,321), and participants who withdrew from the study for unknown reasons (n=10). Ultimately, we included 14,251 participants for data analysis, and the flowchart of participant selection was shown in **Figure 1**. In the previous study, Okamura et al. reported that the NAGALA project obtained approval from the Murakami Memorial Hospital Ethics Committee, and informed consent was obtained from all participants (24). As a post-analysis, the new study protocol obtained approval from the Ethics Committee of Jiangxi Provincial People’s Hospital (IRB2021-066). The entire study process followed the Strengthening the Reporting of Observational Studies in Epidemiology (STROBE) reporting guidelines and the Helsinki Declaration.

### Baseline data collection and definitions

As previously described (24), participants were invited to undergo a baseline assessment, during which trained physicians and nurses collected general demographic information (gender, age), lifestyle information (smoking/drinking status and exercise habits), medical history of chronic diseases, medication history, and baseline physical measurements [height, weight, WC, and arterial blood pressure]. These measurements and evaluations were recorded in standardized questionnaires. Smoking status was categorized based on past smoking history: Non, Past, and Current. Drinking status was categorized based on the reference of weekly alcohol intake in the past month: Non or small (<40 g/week), Light (40-139 g/week), and Moderate (140-209 g/week) (26). Exercise habits were defined as engaging in physical activity at least once a week. Height, weight, WC, and arterial blood pressure were measured indoors using standardized methods, and body mass index (BMI) was calculated from height and weight. Metabolic syndrome (MetS) was defined according to Asian criteria, with any three of the following five components to make a diagnosis of MetS: (1) WC ≥90 cm in males or ≥80 cm in females; (2) TG≥150 mg/dL; (3) HDL-C <40mg/dL in males or <50mg/dL in females; (4) Systolic ≥130 and/or diastolic ≥85mmHg; (5) FPG ≥100mg/dL (27).

Venous blood samples for biochemical analysis were collected from participants after an overnight fasting period. The concentrations of ALT, AST, GGT, HDL-C, total cholesterol (TC), triglyceride (TG), hemoglobin A1c (HbA1c), and FPG were measured using automated analyzers in a standard laboratory. Low density lipoprotein cholesterol (LDL-C) concentration was calculated using the Friedewald equation (28).

### Study outcome

The outcome of our study was assessed as the diagnosis of NAFLD. The evaluation of NAFLD was performed through liver ultrasound scanning, excluding excessive alcohol consumption and other liver disease causes that have been previously confirmed (29). This procedure was carried out by trained ultrasound professionals, and gastroenterology experts made the final diagnosis based on the comprehensive evaluation of ultrasound features, including liver brightness, hepatorenal echo contrast, vascular blurring, and deep attenuation, without referencing other participant information (29).

### Statistical analysis

We described the baseline characteristics of participants using quartiles of the ALT/HDL-C ratio. Chi-square test, one-way ANOVA, and Kruskal-Wallis H test were performed to examine the differences among quartiles of the ALT/HDL-C ratio.

In the logistic regression models, we examined the association between the ALT/HDL-C ratio and NAFLD, and the results were reported as ORs and 95% CIs per SD increment. The establishment of the multivariable models followed the STROBE guidelines and involved a stepwise adjustment process (30). Model 1 focused on the influence of gender, age, height, and BMI. Model 2 further adjusted for smoking and drinking status, as well as exercise habits. Model 3, as the final model, included all non-collinear variables (collinearity among variables was assessed using the variance inflation factor, see **Supplementary Table 1**) (31). It expanded on Model 2 by additionally considering the potential impact of blood glucose, blood pressure, blood lipids, and liver enzyme-related parameters. The same analytical steps were repeated in different subgroups stratified by gender, age, and WC, with the thresholds for age groups referencing WHO standard, and the grouping threshold of WC adopted the Asian standard (abdominal obesity was defined as a WC ≥90 cm in males or ≥80 cm in females as a cut-off) (27, 32). The differences between different strata were further examined using the likelihood ratio test to determine if there were interactions between the ALT/HDL-C ratio and NAFLD with age, gender, and WC.

After establishing the association between the ALT/HDL-C ratio and NAFLD, we further assessed the diagnostic value of the ALT/HDL-C ratio and its components for identifying NAFLD using ROC analysis. The AUCs were calculated, and statistical comparisons were performed using the DeLong test (33).

Finally, to further verify the robustness of the current study, we also compared the correlation of various liver function indexes/HDL-C ratios with NAFLD, including ALT/HDL-C ratio, AST/HDL-C ratio and GGT/HDL -C ratio. These parameters were all Z-transformed to eliminate the effect of dimension, and the results were shown in increments per SD. In addition, based on ROC analysis, we further compared the identification value of these three ratio parameters for NAFLD.

Statistical software R version 3.4.3 and Empower Stats version 2.0 were used for the analysis, with a significance level set at two-sided *P* < 0.05.

## Results

### Baseline characteristics

A total of 14,251 participants were included in the current study, with an average age of 43.53 years and a male-to-female ratio of 1.08. The mean ALT/HDL-C ratio for all participants was 15.43, and the case of NAFLD was 2,507 individuals (17.59%). **Table 1** summarizes the baseline characteristics of the participants according to quartiles of the ALT/HDL-C ratio. It can be observed that participants with higher ALT/HDL-C ratios were more likely to be male, MetS patients (Q1: 0.39% VS Q2 1.34% VS Q3 4.75% VS Q4 19.54%), smokers and drinkers, and had a higher prevalence of NAFLD (Q1: 1.66% VS Q2 4.90% VS Q3 15.31% VS Q4 48.40%). They also had relatively higher values of height, weight, WC, ALT, AST, GGT, TC, TG, LDL-C, FPG, HbA1c, systolic blood pressure, and diastolic blood pressure levels.

**Table 1:** Baseline characteristics of four groups.

	ALT/HDL-C ratio quartile				*P*-value
	Q1(<7.84)	Q2(7.84-11.42)	Q3(11.42-18.07)	Q4(>18.08)	
**No. of subjects**	3552	3569	3559	3562	
**Age, years**	41.00 (36.00-47.00)	42.00 (37.00-50.00)	44.00 (37.00-52.00)	42.00 (36.00-50.00)	<0.001
**Gender**					<0.001
**Female**	2970 (83.61%)	2260 (63.32%)	1229 (34.53%)	381 (10.70%)	
**Male**	582 (16.39%)	1309 (36.68%)	2330 (65.47%)	3181 (89.30%)	
**Weight, kg**	52.73 (7.67)	56.16 (9.00)	61.97 (10.28)	70.17 (10.93)	<0.001
**Height, cm**	160.86 (7.22)	162.90 (8.35)	166.22 (8.59)	169.19 (7.20)	<0.001
**BMI, kg/m^2^**	20.34 (2.26)	21.10 (2.54)	22.36 (2.87)	24.46 (3.13)	<0.001
**WC, cm**	70.37 (6.73)	73.16 (7.43)	77.56 (8.08)	83.64 (8.06)	<0.001
**ALT, U/L**	11.00 (9.00-12.00)	15.00 (13.00-17.00)	19.00 (16.00-21.00)	29.00 (24.00-38.00)	<0.001
**AST, U/L**	14.00 (12.00-17.00)	16.00 (14.00-19.00)	18.00 (15.00-21.00)	22.00 (18.00-27.00)	<0.001
**GGT, U/L**	11.00 (9.00-13.00)	13.00 (10.00-16.00)	16.00 (13.00-21.00)	24.00 (17.00-36.00)	<0.001
**HDL-C, mmol/L**	1.82 (0.37)	1.56 (0.31)	1.34 (0.28)	1.12 (0.25)	<0.001
**TC, mmol/L**	5.06 (0.82)	5.03 (0.85)	5.12 (0.88)	5.28 (0.89)	<0.001
**LDL-C, mmol/L**	2.73 (2.32-3.20)	2.91 (2.46-3.39)	3.14 (2.67-3.65)	3.41 (2.92-3.89)	<0.001
**TG, mmol/L**	0.52 (0.38-0.70)	0.61 (0.43-0.85)	0.81 (0.56-1.14)	1.20 (0.82-1.73)	<0.001
**FPG, mmol/L**	4.99 (0.39)	5.06 (0.40)	5.19 (0.39)	5.35 (0.37)	<0.001
**HbA1c, %**	5.15 (0.30)	5.16 (0.31)	5.17 (0.33)	5.22 (0.34)	<0.001
**SBP, mmHg**	108.51 (13.22)	111.33 (14.27)	115.07 (14.39)	120.80 (14.44)	<0.001
**DBP, mmHg**	67.03 (9.42)	69.21 (9.74)	72.12 (10.05)	76.11 (10.02)	<0.001
**Exercise habits**	559 (15.74%)	654 (18.32%)	720 (20.23%)	535 (15.02%)	<0.001
**Drinking status**					<0.001
**Non or small**	3145 (88.54%)	3032 (84.95%)	2828 (79.46%)	2797 (78.52%)	
**Light**	342 (9.63%)	389 (10.90%)	501 (14.08%)	522 (14.65%)	
**Moderate**	65 (1.83%)	148 (4.15%)	230 (6.46%)	243 (6.82%)	
**Smoking status**					<0.001
**Non**	2855 (80.38%)	2526 (70.78%)	1897 (53.30%)	1464 (41.10%)	
**Past**	368 (10.36%)	500 (14.01%)	778 (21.86%)	910 (25.55%)	
**Current**	329 (9.26%)	543 (15.21%)	884 (24.84%)	1188 (33.35%)	
**MetS**	14 (0.39%)	48 (1.34%)	169 (4.75%)	696 (19.54%)	<0.001
**NAFLD**	59 (1.66%)	175 (4.90%)	545 (15.31%)	1724 (48.40%)	<0.001

Values were expressed as mean (standard deviation) or medians (quartile interval) or n (%). For continuous variables, we employed one-way ANOVA and the Kruskal-Wallis H test to analyze whether there were differences in means/medians among ALT/HDL-C quartile groups, thus assessing the effect of ALT/HDL-C ratio grouping. For categorical variables, we used the chi-square test to analyze whether there were differences in the frequencies (%) among ALT/HDL-C quartiles, thereby evaluating the effect of ALT/HDL-C ratio grouping.

BMI, body mass index; WC, waist circumference; ALT, alanine aminotransferase; AST, aspartate aminotransferase; GGT, gamma-glutamyl transferase; HDL-C, high-density lipoprotein cholesterol; TC, total cholesterol; LDL-C, low density lipoprotein cholesterol; TG, triglyceride; HbA1c, hemoglobin A1c; FPG, fasting plasma glucose; SBP, systolic blood pressure; DBP, diastolic blood pressure; ALT/HDL-C ratio, alanine aminotransferase to high-density lipoprotein cholesterol ratio; MetS, metabolic syndrome; NAFLD, non-alcoholic fatty liver disease.

### Association between ALT/HDL-C ratio and NAFLD

Logistic regression analysis showed a significant positive association between the ALT/HDL-C ratio and NAFLD when the ALT/HDL-C ratio was treated as a continuous variable in the unadjusted model, stepwise adjusted models (models 1 and 2), and fully adjusted model (model 3) (**Table 2**). Based on the results from model 3, the OR for the association between the ALT/HDL-C ratio and NAFLD was 3.05 (95% CI: 2.63, 3.53). When the ALT/HDL-C ratio was treated as a categorical variable, we observed a gradual increase in the OR for NAFLD with increasing ALT/HDL-C ratio quartiles in both adjusted and unadjusted models, indicating a positive trend (All *P*-trend < 0.0001). Furthermore, we further analyzed the relationship between AST/HDL-C ratio, GGT/HDL-C ratio and NAFLD. The results showed that the ALT/HDL-C ratio was more strongly associated with NAFLD in all models than the AST/HDL-C ratio and GGT/HDL-C ratios; This finding suggested that the ALT/HDL-C ratio may be a better indicator of NAFLD risk assessment.

**Table 2:** Logistic regression analyses for the association between ALT/HDL-C ratio, AST/HDL-C ratio, GGT/HDL-C ratio and NAFLD.

	Odds ratios (95% confidence interval)			
	Unadjusted model	Model 1	Model 2	Model 3
**ALT/HDL-C ratio (Per SD increase)**	8.61 (7.80, 9.51)	3.30 (2.96, 3.67)	3.23 (2.90, 3.60)	3.05 (2.63, 3.53)
**AST/HDL-C ratio (Per SD increase)**	5.47 (4.99, 6.00)	2.02 (1.82, 2.23)	1.96 (1.77, 2.17)	1.45 (1.30, 1.62)
**GGT/HDL-C ratio (Per SD increase)**	2.41 (2.29, 2.53)	1.38 (1.31, 1.45)	1.43 (1.35, 1.50)	1.20 (1.14, 1.27)
ALT/HDL-C ratio (Quartile)				
**Quartile 1**	Ref	Ref	Ref	Ref
**Quartile 2**	3.05 (2.26, 4.12)	2.01 (1.47, 2.75)	1.99 (1.46, 2.73)	1.83 (1.34, 2.51)
**Quartile 3**	10.71 (8.15, 14.07)	4.39 (3.27, 5.89)	4.32 (3.22, 5.80)	3.68 (2.73, 4.96)
**Quartile 4**	55.53 (42.58, 72.42)	14.60 (10.87, 19.61)	14.34 (10.66, 19.28)	9.96 (7.32, 13.57)
***P*-trend**	<0.0001	<0.0001	<0.0001	<0.0001

ALT/HDL-C ratio: alanine aminotransferase to high-density lipoprotein cholesterol ratio; SD: standard deviation; NAFLD: non-alcoholic fatty liver disease.

Model 1 adjusted for age, sex, height and BMI;

Model 2 adjusted for age, sex, height, BMI, exercise habits, smoking status and drinking status;

Model 3 adjusted for age, sex, height, BMI, exercise habits, smoking status, drinking status, AST, GGT, TC, TG, FPG, HbA1c and SBP.

In the analysis of AST/HDL-C ratio and GGT/HDL-C ratio and NAFLD in Model 3, the variables themselves were not adjusted.

### Subgroup analysis

**Table 3** summarizes the results of the association between the ALT/HDL-C ratio and NAFLD in subgroups stratified by gender, age, and WC. It is worth noting that higher prevalence of NAFLD was observed in the subgroup of individuals aged 44 to 59 years (20.35%), males (27.41%), and abdominal obesity (48.73%). After adjusting for variables in model 3 across all subgroups, a stronger association between the ALT/HDL-C ratio and NAFLD was observed in the subgroup of individuals aged 45 years and above, males, and abdominal obesity. Furthermore, likelihood ratio tests revealed significant interactions between these common subgroups and the association (All *P*-interaction < 0.05).

**Table 3:** Stratified associations between ALT/HDL-C ratio and NAFLD by age, gender, WC and exercise habits.

Subgroup	No. of participants	No. of cases	unadjusted OR (95%CI)	adjusted 0R (95%CI)	*P-*interaction
**Age (years)**					0.0106
**18-44**	8296	1319 (15.90%)	7.66 (6.79, 8.66)	2.58 (2.20, 3.03)	
**45-59**	5311	1081 (20.35%)	11.22 (9.40, 13.39)	4.09 (3.29, 5.09)	
**≥60**	635	107 (16.85%)	11.56 (6.21, 21.50)	4.62 (2.48, 8.60)	
**Gender**					<0.0001
**Female**	6840	478 (6.99%)	4.22 (3.28, 5.43)	1.18 (1.08, 1.30)	
**Male**	7402	2029 (27.41%)	7.31 (6.49, 8.24)	3.56 (3.05, 4.16)	
**Abdominal obesity**					0.0236
**Yes**	1853	903 (48.73%)	1.10 (1.09, 1.11)	1.06 (1.05, 1.08)	
**No**	12389	950 (7.67%)	1.10 (1.10, 1.11)	1.05 (1.04, 1.06)	

ALT/HDL-C ratio, alanine aminotransferase to high-density lipoprotein cholesterol ratio; OR, Odds ratios; CI, confidence interval; BMI, body mass index.

Models adjusted for the same covariates as in model 3 (**Table 2**), except for the stratification variable; Abdominal obesity was defined as a waist circumference ≥90 cm in meles or ≥80 cm in females as a cut-off.

### ROC analysis

**Figure 2:**
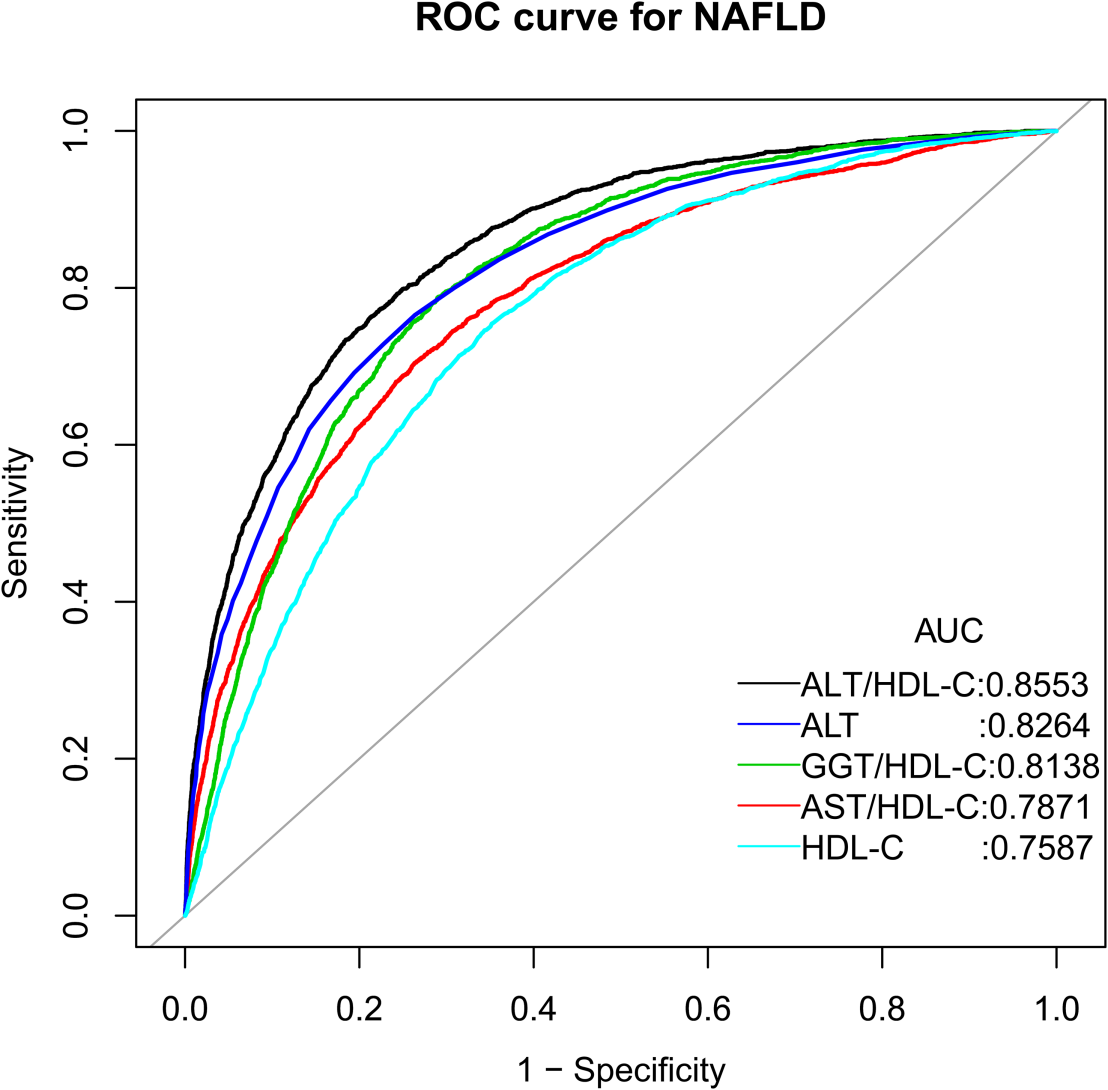
The ROC curve of the diagnostic efficiency of ALT, HDL-C and ALT/HDL-C ratio. AUC: area under the curve; ALT/HDL-C ratio: alanine aminotransferase to high-density lipoprotein cholesterol ratio; AST/HDL-C ratio: aspartate aminotransferase to HDL-C ratio; GGT/HDL-C ratio: gamma-glutamyl transferase to HDL-C ratio.

By plotting the ROC curves, we calculated the AUC, optimal threshold, sensitivity, and specificity for the ALT/HDL-C ratio, AST/HDL-C ratio, GGT/HDL-C ratio, ALT and HDL-C in identifying NAFLD (**Table 4**; **Figure 2**). The results showed that the ALT/HDL-C ratio had the highest AUC (0.8553), and specificity (0.7872) in identifying NAFLD. Furthermore, the Delong test indicated that compared to ALT, HDL-C, AST/HDL-C ratio and GGT/HDL-C ratio, the ALT/HDL-C ratio significantly improved the discriminatory performance for NAFLD at a statistical level.

**Table 4:** Areas under the receiver operating characteristic curves for each evaluated parameter in identifying NAFLD.

	AUC	95%CI low	95%CI upp	Best threshold	Specificity	Sensitivity
**ALT/HDL-C ratio**	0.8553	0.8473	0.8632	15.9700	0.7872	0.7627
AST/HDL-C ratio*	0.7871	0.7771	0.7970	14.3695	0.7365	0.7044
GGT/HDL-C ratio*	0.8138	0.8052	0.8223	12.9311	0.7105	0.7875
ALT*	0.8264	0.8174	0.8353	19.5000	0.7361	0.7655
HDL-C*	0.7587	0.7489	0.7685	1.3408	0.6481	0.7543

AUC: area under the curve; other abbreviations as in **Table 1**.

*P<0.001, compare with ALT/HDL-C ratio (Delong test).

## Discussion

In this study, we examined the association between the ALT/HDL-C ratio and NAFLD and conducted further stratified analyses to determine a significantly stronger association between the two in individuals aged ≥45 years, males, and those who were abdominal obesity. Additionally, the ROC analysis showed a significant improvement in the ability to identify NAFLD using the ALT/HDL-C ratio (AUC=0.8553) compared to ALT, HDL-C, AST/HDL-C ratio and GGT/HDL-C ratio. These findings suggested that the ALT/HDL-C ratio may serve as a potential effective and simple biomarker for evaluating the risk of NAFLD.

Previous studies have extensively investigated the association between ALT or HDL-C individually and NAFLD. It is well established that low levels of HDL-C are a risk factor for NAFLD (20, 21), and elevated ALT levels significantly increase NAFLD risk, even within the normal range for ALT (12–15, 34). In our current study, based on the results of ROC analysis, we recommend an ALT threshold of 19.5 U/L for identifying NAFLD, which falls within the currently defined normal range. It is important to pay more attention to assessing or identifying NAFLD in individuals with normal range ALT, as the general population may not be concerned about biochemical parameters within the normal range and may selectively ignore potential risks of chronic diseases. These findings warranted prompt attention and reconsideration of the normal limits of ALT, which may be useful (14, 35–39).

The ALT/HDL-C ratio, as a combined parameter of ALT and HDL-C, is currently mainly explored in clinical research (22). From a reverse perspective, setting the threshold for the ALT/HDL-C ratio usually requires accumulating sufficient clinical research evidence, which largely avoids selectively ignoring the risk of chronic diseases due to setting excessively high thresholds. Furthermore, whether in the diabetes study conducted by Cao et al. (22) or in our current NAFLD study, these findings revealed that the ALT/HDL-C ratio significantly improved the identification/prediction ability of chronic diseases compared to ALT or HDL-C alone. In addition, in the current study, we have further compared the value of various liver enzyme indicators/HDL-C ratios in identifying NAFLD; The results showed that ALT/HDL-C ratio was significantly better than AST/HDL-C ratio and GGT/HDL-C ratio in identifying NAFLD. These results collectively suggested that combining ALT and HDL-C may be useful for monitoring or diagnosing chronic diseases. According to the results of the ROC analysis of the current study, an ALT/HDL-C ratio of 15.97 was suggested as a screening threshold for identifying NAFLD.

The meaningful findings from subgroup analyses deserve attention in this study. We differentiated several common phenotypes based on age, gender, and WC. The results of the subgroup analysis revealed a stronger association between ALT/HDL-C ratio and NAFLD in individuals aged ≥45 years, males, and those who were abdominal obesity. This suggested that individuals in these subgroups may be at a higher risk for NAFLD, and monitoring the ALT/HDL-C ratio could be useful for them. We have made some wild guesses as to why the correlation between the ALT/HDL-C ratio and NAFLD was stronger in these populations in particular, based on the results of the current study and some published studies. Firstly, when considering the composition of ALT/HDL-C ratio, it has been observed that males and abdominal obesity individuals often have higher ALT levels (40, 41) and lower HDL-C levels (42–45) compared to females and normal-weight individuals. These gender and obesity-related differences directly contribute to higher baseline ALT/HDL-C ratios in males and abdominal obesity individuals, resulting in a relatively higher risk of NAFLD. However, in age-specific studies, it is not possible to directly explain the relatively higher risk of NAFLD by analyzing the individual components of the ALT/HDL-C ratio. This is because, with increasing age, the levels of ALT showed an inverse dose-response relationship (46, 47), while HDL-C levels tended to remain relatively stable and not undergo significant changes with age (45, 48, 49). Secondly, let’s continue the analysis based on population characteristics. Generally, older individuals, males, and abdominal obesity individuals tend to have poorer metabolic outcomes (50–52), and in the current study, we found that the proportion of MetS patients among these people was much higher (**Supplementary Table 2**). Therefore, the higher risk of NAFLD associated with the ALT/HDL-C ratio in these populations may be mediated through other metabolic pathways that contribute to these unfavorable outcomes. Lastly, the specific biological activities, functional states, and potential interactions between ALT and HDL-C in the population (53–55) may partially explain the association between ALT/HDL-C ratio and NAFLD in specific populations. However, further research is needed to validate these findings and hypotheses.

Further contemplation of the research findings may provide clearer directions for future studies. To date, there has been only one published study exploring the application of the ALT/HDL-C ratio in predicting diabetes (22). The current study, as an extension of Cao et al.’s research, demonstrated for the first time the potential utility of the ALT/HDL-C ratio in identifying NAFLD (AUC=0.8553). From a disease perspective, both NAFLD and diabetes are metabolic disorders that affect multiple organs in the body (5, 6, 56). They share common mechanisms involving IR (57, 58), and obesity is a major risk factor for both diseases (1, 2, 57, 58). Therefore, future research could focus more on the association between the ALT/HDL-C ratio and IR, metabolic disorders, and obesity-related diseases to further validate its applicability in other conditions. From a biomarker perspective, ALT is a key enzyme indicative of liver function (8), while HDL-C primarily functions in the liver (16, 17). The ALT/HDL-C ratio, as a combined index of both, may also provide assistance in more accurate assessment of liver function and the risk of other liver diseases.

### Strengths and limitations

The main strength of our study lies in the utilization of a large sample size, which allowed us to uncover the association between the ALT/HDL-C ratio and NAFLD for the first time. These findings provided a valuable biomarker for the screening of NAFLD and served as important references for future research.

Our study also has some limitations that need to be addressed. Firstly, regarding the study design, our study employed a cross-sectional design, which does not allow us to establish causal relationships. Secondly, in terms of the evaluation of study outcomes, the diagnosis of NAFLD in our study was based on liver ultrasonography after excluding the influences of other liver diseases and alcohol consumption, which may have resulted in the omission of individuals with mild hepatic steatosis (59). Thirdly, concerning data analysis, although we conducted rigorous statistical adjustments in the current analysis, there might still be some unaccounted confounding factors leading to residual confounding. Lastly, regarding the generalizability of the study findings, our study was based on data from participants in the Japanese NAGALA project. Therefore, further validation research is needed to extend the findings of our study to other ethnic populations.

## Conclusion

The results of this study indicate a positive association between the ALT/HDL-C ratio and NAFLD. Furthermore, the ALT/HDL-C ratio was found to be effective in identifying NAFLD with a suggested threshold of 15.97.

## Data availability statement

The datasets presented in this study can be found in online repositories. The names of the repository/repositories and accession number(s) can be found in the article/**Supplementary Material**.

## Ethics statement

The studies involving humans were approved by the Ethics Committee of Jiangxi Provincial People’s Hospital. The studies were conducted in accordance with the local legislation and institutional requirements. Written informed consent for participation was not required for this study in accordance with the national legislation and the institutional requirements.

## Author contributions

YZ, GS, JQ, MK and RY conceived the research, drafted the manuscript, and did the statistical analysis. YZ revised the manuscript and designed the study. All authors contributed to the article and approved the submitted version.
