# Supplementary material for: The newly proposed alanine aminotransferase to high-density lipoprotein cholesterol ratio has shown effectiveness in identifying non-alcoholic fatty liver disease
Source: Front Endocrinol (Lausanne). 2023 Aug 31;14:1239398. doi: 10.3389/fendo.2023.1239398 (PMC10505795; doi:10.3389/fendo.2023.1239398)
Supplement: Supplementary file 2 [file Table_2.docx]

Supplementary Table 1: Collinearity diagnostics steps.

|  | Step 1 | Step 2 | Step 3 | Step 4 | Step 5 | Step 6 | Step 7 |
| --- | --- | --- | --- | --- | --- | --- | --- |
| ALT/HDL-C ratio | 5.7 | 5.7 | 5.7 | 5.2 | 2.9 | 2.9 | 2.9 |
| Sex | 3.3 | 3.3 | 3.3 | 3.2 | 3.1 | 3.1 | 3.1 |
| Age | 1.4 | 1.4 | 1.4 | 1.4 | 1.4 | 1.3 | 1.3 |
| Weight | 168.6 | 168.6 | NA | NA | NA | NA | NA |
| Height | 51.9 | 51.9 | 2.9 | 2.9 | 2.9 | 2.4 | 2.4 |
| BMI | 95.9 | 95.9 | 5 | 5 | 4.9 | 1.5 | 1.5 |
| WC | 5.9 | 5.9 | 5.9 | 5.9 | 5.9 | NA | NA |
| Exercise habits | 1 | 1 | 1 | 1 | 1 | 1 | 1 |
| ALT | 7.7 | 7.7 | 7.6 | 7.4 | NA | NA | NA |
| AST | 3.5 | 3.5 | 3.5 | 3.5 | 2.8 | 2.8 | 2.8 |
| GGT | 1.5 | 1.5 | 1.5 | 1.5 | 1.4 | 1.4 | 1.4 |
| HDL-C | Inf | NA | NA | NA | NA | NA | NA |
| TC | Inf | 8.4 | 8.4 | 1.3 | 1.3 | 1.3 | 1.3 |
| LDL-C | Inf | 8.7 | 8.7 | NA | NA | NA | NA |
| TG | Inf | 1.5 | 1.5 | 1.5 | 1.5 | 1.5 | 1.5 |
| FPG | 1.5 | 1.5 | 1.5 | 1.5 | 1.5 | 1.5 | 1.5 |
| HbA1c | 1.2 | 1.2 | 1.2 | 1.2 | 1.2 | 1.2 | 1.2 |
| SBP | 5.5 | 5.5 | 5.5 | 5.5 | 5.5 | 5.5 | 1.4 |
| DBP | 5.6 | 5.6 | 5.6 | 5.6 | 5.6 | 5.6 | NA |
| Drinking status | 1.2 | 1.2 | 1.2 | 1.2 | 1.2 | 1.2 | 1.2 |
| Smoking status | 1.4 | 1.4 | 1.4 | 1.4 | 1.4 | 1.4 | 1.4 |

Abbreviations as in Table ​1.

Note-1: Variance inflation factor = 1/(1-R^2^). Abbreviations as in Table 1.

Note-2: The variables with Variance inflation factor >5 will be regarded as collinear variables and cannot be included in the multiple regression model.

Supplementary Table 2: Summarize whether the population has Metabolic syndrome according to subgroup classification.

|  | Metabolic syndrome | | *P*-value |
| --- | --- | --- | --- |
|  | No | Yes |  |
| Age (years) |  |  | <0.001 |
| 18-44 | 7847 (58.91%) | 455 (48.92%) |  |
| 45-59 | 4903 (36.81%) | 411 (44.19%) |  |
| ≥60 | 571 (4.29%) | 64 (6.88%) |  |
| Gender |  |  | <0.001 |
| Female | 6610 (49.62%) | 230 (24.73%) |  |
| Male | 6711 (50.38%) | 700 (75.27%) |  |
| Abdominal obesity |  |  | <0.001 |
| Yes | 12070 (90.61%) | 328 (35.27%) |  |
| No | 1251 (9.39%) | 602 (64.73%) |  |

Abdominal obesity was defined as a waist circumference ≥90 cm in males or ≥80 cm in females as a cut-off
